# Plant–animal interactions between carnivorous plants, sheet‐web spiders, and ground‐running spiders as guild predators in a wet meadow community

**DOI:** 10.1002/ece3.6230

**Published:** 2020-03-30

**Authors:** James J. Krupa, Kevin R. Hopper, Samuel B. Gruber, Jason M. Schmidt, James D. Harwood

**Affiliations:** ^1^ Department of Biology University of Kentucky Lexington KY USA; ^2^ Biological Sciences Bluegrass Community and Technical College Lexington KY USA; ^3^ Department of Entomology University of Kentucky Lexington KY USA; ^4^ Present address: Department of Entomology University of Georgia 2360 Rainwater Road Tifton GA 31793 USA; ^5^ Present address: College of Plant Health and Medicine Qingdao Agricultural University 700 Changcheng Road Qingdao Shandong 266109 China

**Keywords:** amensalism, asymmetrical competition, carnivorous plants, dwarf sundews, exploitative competition, ground‐running spiders, interkingdom competition, plant–animal interactions, sheet‐web spiders

## Abstract

1. Plant–animal interactions are diverse and widespread shaping ecology, evolution, and biodiversity of most ecological communities. Carnivorous plants are unusual in that they can be simultaneously engaged with animals in multiple mutualistic and antagonistic interactions including reversed plant–animal interactions where they are the predator. Competition with animals is a potential antagonistic plant–animal interaction unique to carnivorous plants when they and animal predators consume the same prey.
2. The goal of this field study was to test the hypothesis that under natural conditions, sundews and spiders are predators consuming the same prey thus creating an environment where interkingdom competition can occur.
3. Over 12 months, we collected data on 15 dates in the only protected Highland Rim Wet Meadow Ecosystem in Kentucky where sundews, sheet‐web spiders, and ground‐running spiders co‐exist. One each sampling day, we attempted to locate fifteen sites with: (a) both sheet‐web spiders and sundews; (b) sundews only; and (c) where neither occurred. Sticky traps were set at each of these sites to determine prey (springtails) activity–density. Ground‐running spiders were collected on sampling days. DNA extraction was performed on all spiders to determine which individuals had eaten springtails and comparing this to the density of sundews where the spiders were captured.
4. Sundews and spiders consumed springtails. Springtail activity–densities were lower, the higher the density of sundews. Both sheet‐web and ground‐running spiders were found less often where sundew densities were high. Sheet‐web size was smaller where sundew densities were high.
5. The results of this study suggest that asymmetrical exploitative competition occurs between sundews and spiders. Sundews appear to have a greater negative impact on spiders, where spiders probably have little impact on sundews. In this example of interkingdom competition where the asymmetry should be most extreme, amensalism where one competitor experiences no cost of interaction may be occurring.

## Graphical abstract

This field study examined the plant–animal interactions between a carnivorous plant (dwarf sundew) and both sheet‐web spiders and ground‐running spiders. Both the plants and the spiders function as predators of the same springtail prey. Thus, the potential for competition exists. The results of this study suggest that interkingdom, exploitative competition is occurring. Furthermore, this is asymmetrical competition where the sundews experience little negative impact to the extent of this being an example of amensalism.

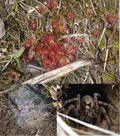

## 1 INTRODUCTION

Plant–animal interactions are diverse and widespread shaping ecology, evolution, and biodiversity of most ecological communities (Giron et al., 2018; Herrera et al., 2002; Lewinsohn, Prado, Jordano, Bascompte, & Olesen, 2006; Strauss & Irwin, 2004). Most angiosperms are influenced by interactions with animals in some form (Steele, Yi, & Zhang, 2018). These interactions can be complex where plants have different, simultaneous interactions with multiple animals ranging from weak to strong and occurring along a mutualistic‐antagonistic gradient (Rodríguez‐Rodríguez, Jordano, & Valido, 2017). Mutualistic relationships favoring both plant and animal, including pollination and seed dispersal, are extremely widespread. Antagonistic relationships are typically a cost to the plant and include herbivory and seed predation.

Charles Darwin (1875) first demonstrated that carnivorous plants capture prey as a source of nutrients. Consequently, these plants have unique and complex plant–animal interactions because ecologically they function as predators. Since they have negative impact on animals, carnivorous plants capturing animals are considered examples of reversed plant–animal interactions (Thompson, 1981) for being the opposite of typical plant–herbivore interactions. Carnivorous plants experience additional plant–animal interactions that other angiosperms do not. These include prey–pollinator conflict where capturing potential pollinators can reduce growth and reproduction (Ellison & Gotelli, 2009), digestive mutualism where animals help carnivorous plants acquire nutrients from prey (Anderson, 2005; Anderson & Midgley, 2003; Chin, Moran, & Clarke, 2011; Ellis & Midgley, 1996; Grafe, Schöner, Kerth, Junaidl, & Schöner, 2011; Lam, Lim, Wong, & Tan, 2018; Lim, Lam, & Tan, 2018; Scharmann, Thornham, Grafe, & Federle, 2013; Schöner et al., 2017), and antagonistic plant–animal interactions such as kleptoparasitism (Burbridge, 1880; Scharmann et al., 2013).

Relatively little work has focused on antagonistic plant–animal interactions where carnivorous plants and animals compete as predators, despite competition between kingdoms possibly being the most common form of competition (Barnes, 2003; Hochberg & Lawton, 1990; Trienens, Keller, & Rohlfs, 2010; Trienens & Rohlfs, 2011). Jennings, Krupa, Raffel, and Rohr (2010) conducted a laboratory experiment and field study suggesting wolf spiders and sundews compete, while Jennings, Krupa, and Rohr (2016) suggested sundews, wolf spiders, and toads compete. Clearly more extensive field studies are needed to understand the dynamics of plant–animal interactions between carnivorous plants and spiders where they co‐exist as predators.

In this field study, we examined interactions between dwarf sundews (*Drosera brevifolia*), sheet‐web‐building spiders (families Hahniidae and Linyphiidae) hereafter referred to as sheet‐web spiders and nonweb‐building wolf spiders (family Lycosidae), hereafter referred to as ground‐running spiders (Uetz, Halaj, & Cady, 1999). We tested the following hypothesis: Under natural conditions, sundews and spiders consume the same prey creating the potential for interkingdom competition.

## 2 STUDY SYSTEM

The dwarf sundew (*D. brevifolia*) has one of the widest distributions of any carnivorous plant in the western hemisphere ranging from North America to South America (United States, Cuba, Mexico, Belize, Brazil, and Uruguay; Correa & dos Santos Silva, 2005; Schnell, 2002). In North America, the distribution is a coastal band that extends from east Texas to Virginia with disjunct populations in Oklahoma, Arkansas, Alabama, Kansas, Kentucky, and Tennessee. The Kentucky population is the northern most of these and is state‐endangered. This population grows in a 0.81‐hectare area in Hazeldell Meadow, Pulaski County, Kentucky. This site is the only remaining, protected Highland Rim Wet Meadow ecosystem left in the state. The associated Robertsville series soil is deep and poorly drained as a result of an underlying fragipan, which creates a shallow water table just beneath the surface. Most sundews grow in a 600 m^2^ portion of the meadow where the soil is compressed and depressed. The population fluctuates greatly from year to year and from season to season. Over a 10‐year period, the population has varied from 220,000 to 25,000 plants. This population is comprised of biennial and perennial sundews the proportions of which vary from year to year depending on temperature and precipitation (Krupa, 2019).

The dwarf sundew, like most carnivorous plants, depends on disturbance in the form of prescribed fire and bare soil brought to the surface by burrowing crayfish (*Cambarus* sp.). Spiders are both diverse and extremely abundant in the meadow and are in close association with sundews. These include wolf spiders (Lycosidae) of the genera *Pirata*, *Allocosa*, *Pardosa*, *Schizocosa*, and *Rabidosa*. Sheet‐web species of the families Linyphiidae and Hahniidae are also abundant and include the genera *Neoantistea*, *Tennessellum*, and *Grammonota*.

## 3 METHODS

### 3.1 Field sampling

This study focused on the largest of the subpopulations of *D. brevifolia* growing in the meadow. Ten 400 cm^2^ plots were established in July 2011. Each plot was counted periodically over the duration of this study. From August 2012 to August 2013, the study site was visited on 15 days over the seasons weather permitting; during significant snow cover, heavy rain, and standing water, data collection was not possible. We systematically walked along a transect on the eastern edge of the study site (where the densest patches of sundews occur), from south to north, identifying all sheet‐webs. After this transect was surveyed, we moved one meter to the west and again walked the length of the sundew population. The goal was to locate 15 sheet‐webs with spiders on each sample date. On some collection days, due to weather, we were unable to find 15 of these webs. Spiders occupying each web were collected with an aspirator, and the location marked and identified with a numbered flag. Each spider was preserved in a separate 1.5 ml microcentrifuge tube filled with 100% EtOH and maintained on ice. Spiders were transferred to a −20°C freezer upon return to the laboratory. The area of a web was calculated by measuring the longest horizontal facial dimension and the dimension perpendicular to it, then calculating an ellipse with these two measures as the radii (Hesselberg, 2010; Welch, 2013). The shape of a web was calculated by dividing length by width.

A 40 cm^2^ metal frame subdivided into a string grid of 100 units was placed on the ground at each collection site with the flag at center. The percent of grid units with at least one sundew was used as a measure of percent cover. The distance and diameter of the three nearest sundews from the site of a sheet‐web were recorded.

In addition to sites where sheet‐web spiders were collected (henceforth referred to as spider sites), 15 sites with sundews that lacked sheet‐webs (sundew sites) were randomly selected and flagged. Percent sundew cover was also recorded for each sundew site. Additionally, 15 sites that lacked sheet‐webs and sundews (control sites) were randomly selected and flagged. After which, circular sticky traps were placed at each site using 60 mm dia. pieces of clear transparency sprayed with Tangle‐Trap Insect Trap Coating Spray (The Tanglefoot Company). Each of these was placed on top of 60‐mm‐dia. Petri dishes painted with dark brown water paint and depressed into the ground to be flushed with the soil surface (modified from sampling protocol described by Harwood, Sunderland, & Symondson, 2001, 2003). These traps were collected after 24 hr, immediately put on ice for transport to the laboratory and placed in a laboratory freezer until all captured arthropods were identified and counted.

On each sampling day, 8–20 ground‐running spiders were collected. Percent sundew cover and both distance and diameter of the three nearest sundews from the point where a ground‐running spider was captured were recorded. Sticky traps were not set out at capture sites of ground‐running spiders, because they were highly mobile ranging over a large area.

Sundews typically grow on open patches of soil where few other angiosperms occur. This required that we determine whether springtails were avoiding open patches (versus being captured by sundews). We set out 30 pairs of 60‐mm‐dia. sticky traps (as described above), one on open areas lacking vegetation and the other in adjacent grass area with dense grass 30 cm away on 3 days (21 October 2012, 30 November 2012, and 30 June 2013). Traps were collected 24 hr later and frozen until captured springtails were counted and identified.

On 3 days (30 May, 15 July, and 29 July 2013), 50 sundew leaves were randomly selected, cut (only one per plant), and individually preserved in a 1.5 ml Eppendorf tubes filled with 100% EtOH. The 150 leaves were kept frozen until captured arthropods were identified.

### 3.2 Direct spider–sundew interactions

A 70‐mm‐dia plastic ring was pressed into the soil surrounding 17 sundews that covered 346 mm^2^ which was 9% of the area within the ring. Individual *Neoantistea agilis* were dropped into the arena one at a time and observed for at least 60 s or until they stopped moving for 60 s. The following behaviors were recorded: (a) runover—a spider ran over a sundew without having sundew mucilage attach to the spider; (b) avoid—a spider approached a sundew with front legs barely touching, stopped, and then moved away; and (c) pull away—a spider ran over a sundew, had sundew mucilage attach to its body then pulled free.

### 3.3 Molecular analysis of predation

Spiders were identified to species, when possible, and whole‐body DNA extractions were performed using QIAGEN DNeasy Tissue Kits (QIAGEN Inc.) following the manufacturer's animal tissue protocol. The DNA extracted from spiders was then screened for the presence of prey DNA using a general Collembola (hereafter referred to as springtails) primer (Chapman, Schmidt, Welch, & Harwood, 2013). PCR procedures, as described by Chapman et al. (2013), were followed which optimized the primers and screened for cross‐reactivity against 155 nonspringtail species. Positive tests for springtails in the diet of spiders were determined by electrophoresis of 10 μl of PCR product in 2% SeaKem agarose (Lonza) stained with 0.1 mg/μl GelRed^™^ (Biotium, Inc.). Even though flies and springtails are most commonly captured by sundews (Ellison & Gotelli, 2009), ground‐dwelling spiders primarily consume springtails (Chapman et al., 2013; Harwood, Sunderland, & Symondson, 2001, 2003). Because of this, springtails are the most likely common prey for sundews and spiders in this study; thus, molecular analysis for flies in spider gut content was not performed.

### 3.4 Statistical analysis

Data were analyzed using R statistical software version 3.6.1 (R Core Team, 2015). We tested for specific predictions of competition, including negative relationships between (i) sundew abundance and shared prey abundance, (ii) spider presence and shared prey abundance, (iii) spider web size and shared prey abundance, (iv) spider presence and sundew abundance, and (v) sundew abundance and spider web size. We used linear mixed‐effects models (lmer function in R) to model predictor variables as fixed effects and sampling date as a random effect to control for the effects of time. ANOVA tables were calculated to compare means between groups. General linear mixed models (glmer function is R) were used when non‐Gaussian data called for a Poisson link function. To ensure estimated coefficients would be on the same scale and facilitate comparisons of effect sizes, explanatory variables were standardized by centering means and scaling standard deviations prior to regression analysis. Chi‐square tests compared the likelihood of spider gut contents containing springtail DNA between the two spider types as well as percent sundew coverage between spiders that had and had not recently consumed springtail prey.

## 4 RESULTS

### 4.1 Sundew population dynamics

**FIGURE 1:**
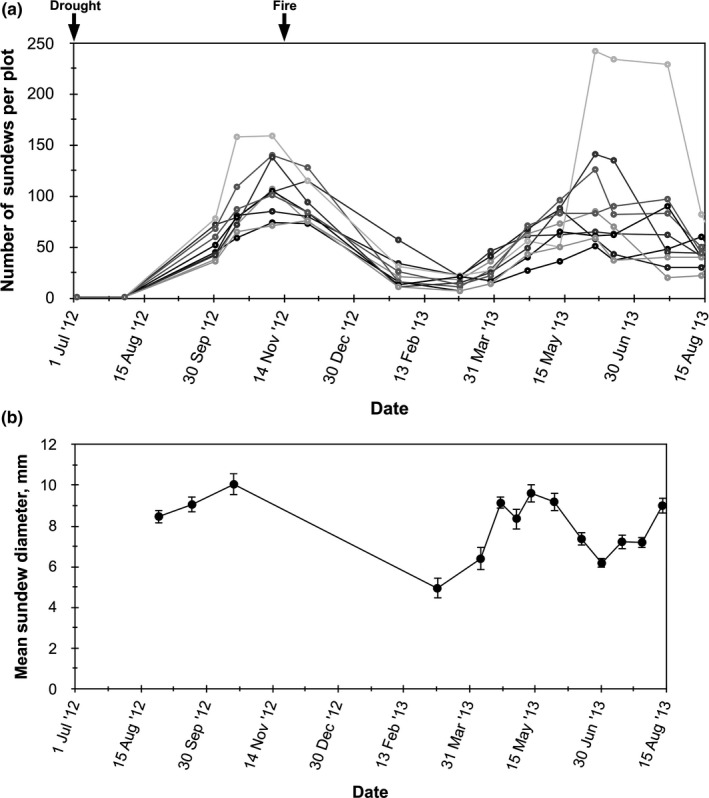
(a) Fluctuation in number of sundews (*Drosera brevifolia*) for each of 10, 400 cm^2^ plots at Hazeldell Meadow from August July 2013 to August 2014; (b) Mean diameter (±1 *SE*) of sundews measured over the study period

By mid‐August 2012 after the end of a 2‐month drought left most adult sundews dead or dormant, sundew seeds began to germinate. Two waves of germination occurred during the fall of 2012 and spring of 2013 (Figure 1a). The site was exposed to a prescribed burn in November 2012, and a wet winter subsequently ensued with standing water typical. Consequently, sundew numbers fluctuated over the 12‐month study (Figure 1a). Sundew plots averaged 1,724 plants/m^2^ during the growing season and 553 plants/m^2^ during winter dormancy from December 2012 to February 2013. Sundews grew rapidly after germinating, and the mean size of plants increased in the fall of 2012 and spring of 2013 (Figure 1b). The smallest mean diameter of plants occurred while they were dormant during the winter.

### 4.2 Spiders captured

A total of 172 sheet‐web spiders were collected during the study belonging to three genera (*Grammonota*, *Neoantistea*, and *Tennessellum*) in two families (Hahniidae and Linyphiidae). *Neoantistea* was the most common genus (71.5%) of sheet‐web spiders collected. A total of 188 ground‐running spiders, all belonging to the family Lycosidae, were collected and represented five genera (*Allocosa*, *Pirata*, *Pardosa*, *Rabidosa*, and *Schizocosa*). *Pardosa* was the most common (71%).

### 4.3 Prey captured by spiders

**FIGURE 2:**
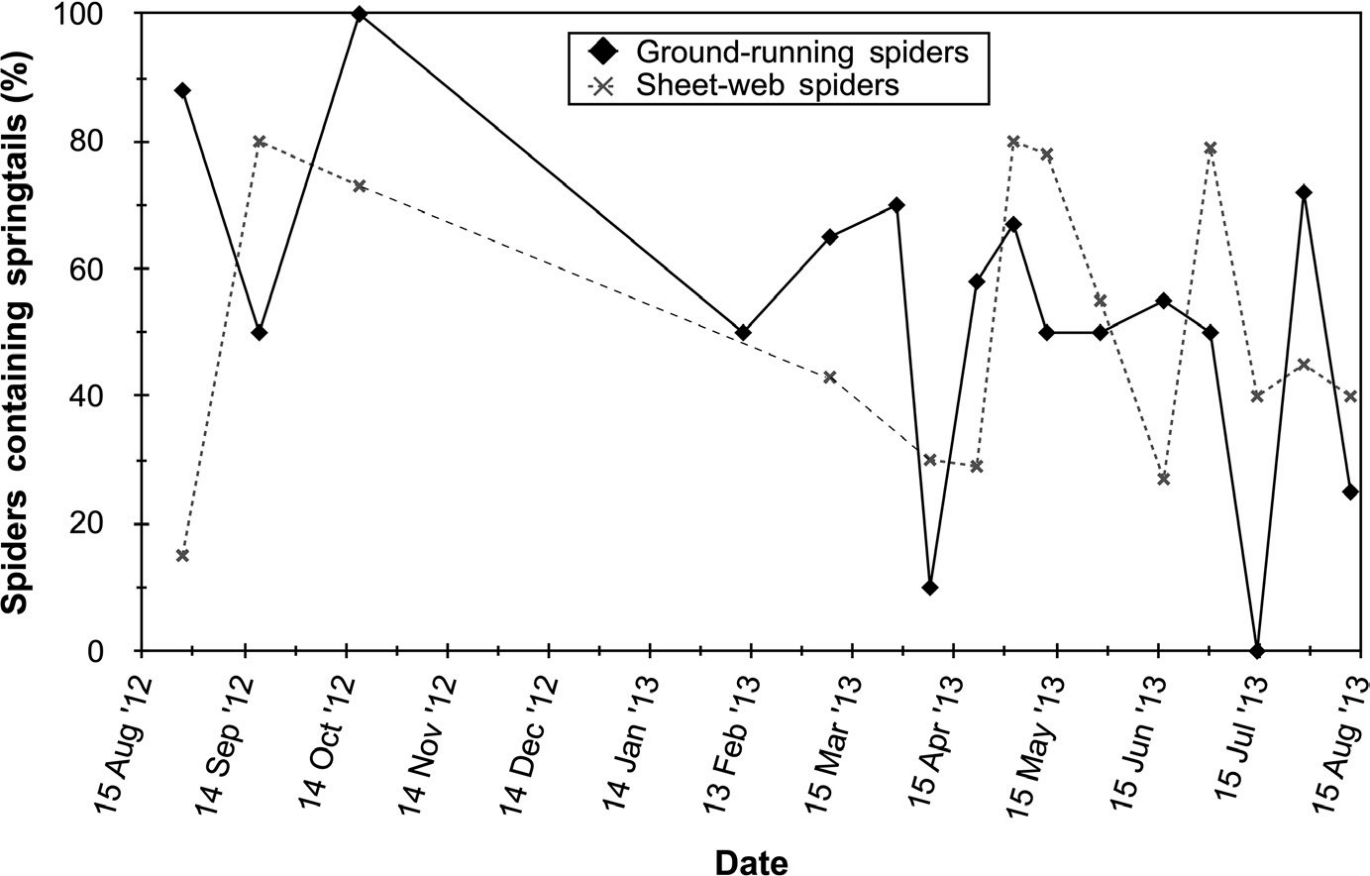
Percentage of captured sheet‐web spiders and ground‐running spiders that contained springtail DNA in their guts for each sampling date

**FIGURE 3:**
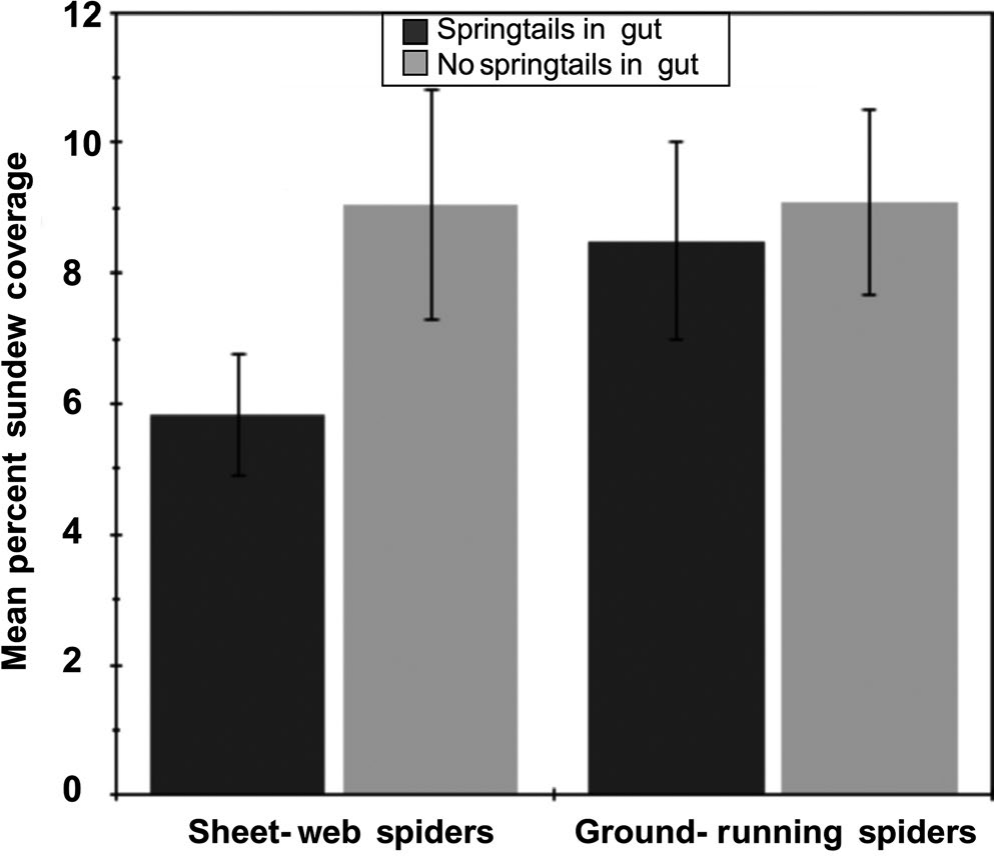
Both sheet‐web spiders and ground‐running spiders with springtail DNA in their gut contents and those without springtail DNA in their guts compared to the percentage of sundew coverage that the site of capture

Molecular analysis of the gut contents of the 360 spiders we collected revealed that 54.9% of sheet‐web spiders and 52.1% of ground‐running spiders tested positive for the presence of springtail DNA, indicating frequent consumption during the period of this study (Figure 2). Sheet‐web spiders testing positive for springtail DNA in their guts were found in areas with significantly lower sundew cover than spiders lacking springtails (*F*_1,151_ = 2.848, *p* = .047, one‐tailed; Figure 3). In contrast, the presence of springtail DNA in the guts of ground‐running spiders did not differ with respect to sundew cover (*F*_1,169_ = 0.083, *p* = .387; Figure 3).

### 4.4 Prey availability to sundews and spiders

Springtails were by far the most common arthropod captured by sticky traps (91% of prey caught). Springtails were also the most common prey trapped on the sundew leaves (40.6%). Springtails were present in the majority of both sheet‐web spider guts (54.9%) and ground‐running spider guts (52.1%).

**FIGURE 4:**
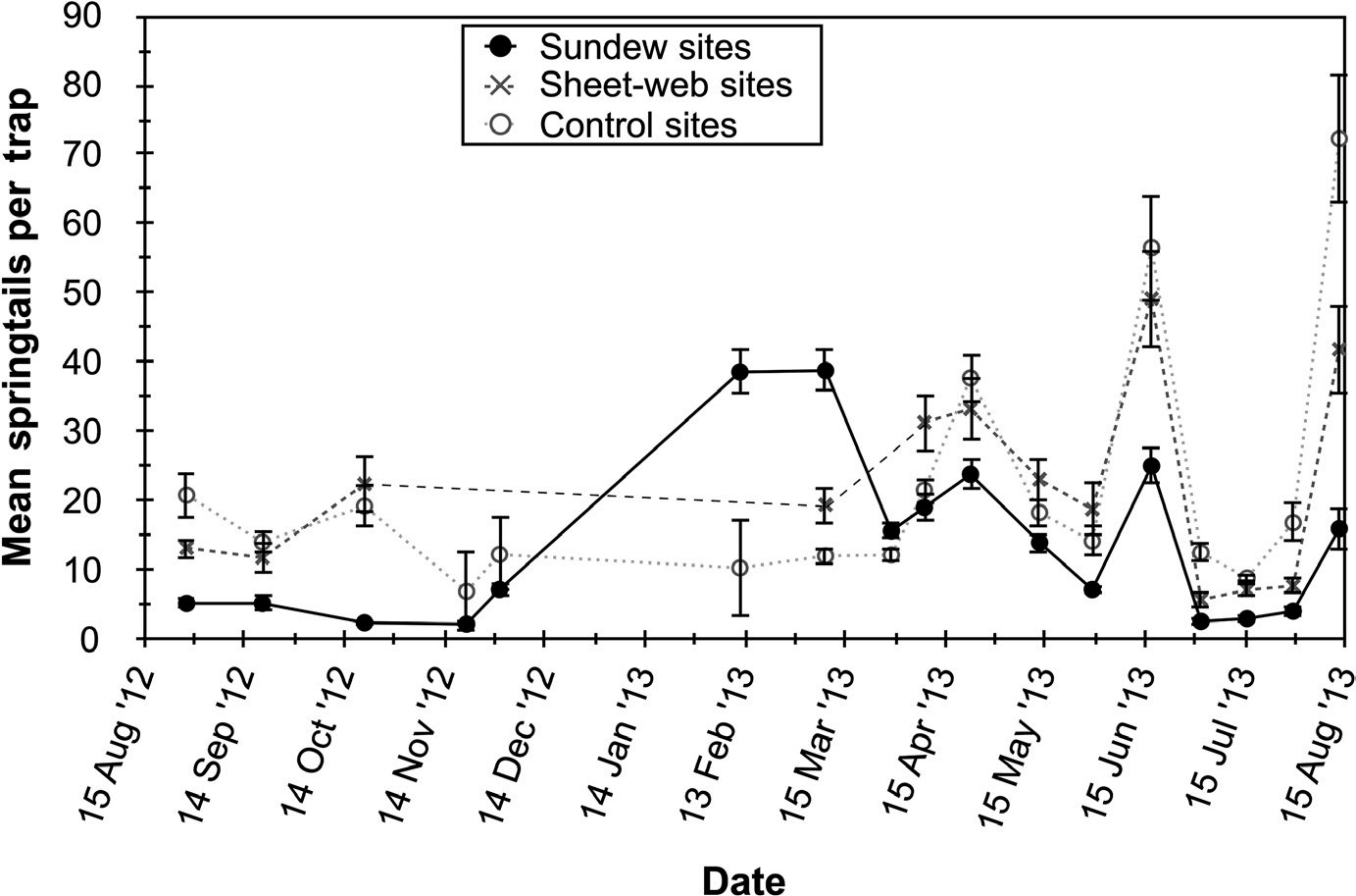
The number of springtails caught on sticky traps placed at sundew, spider, and control sites during the study

**FIGURE 5:**
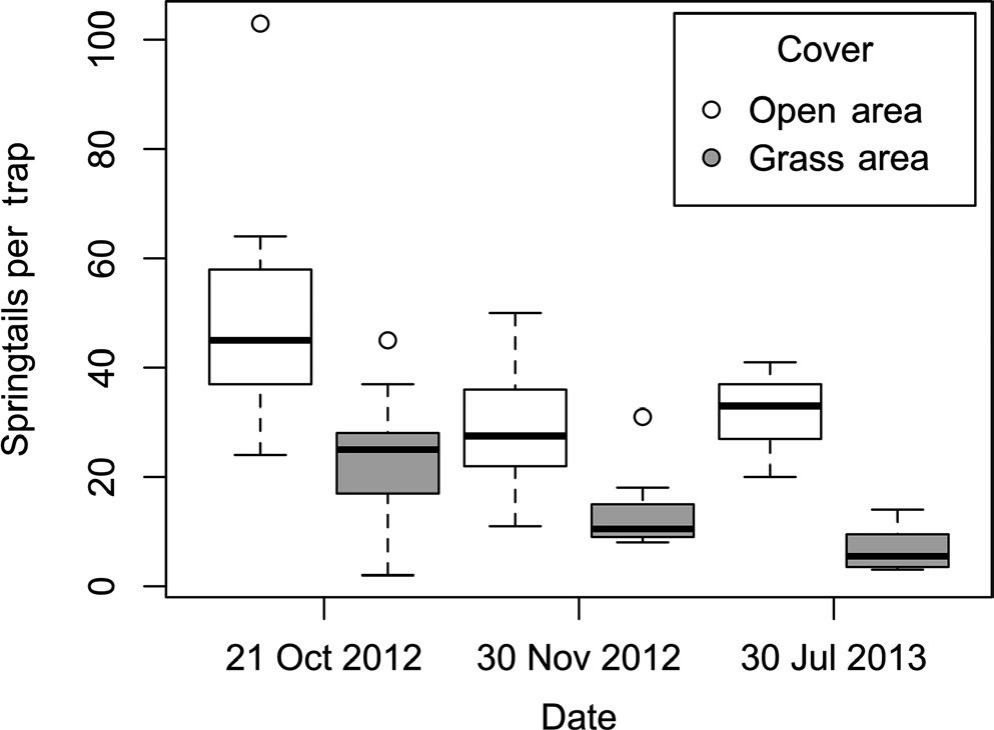
Comparison of sticky traps set out in open versus grass locations overnight on three dates

**FIGURE 6:**
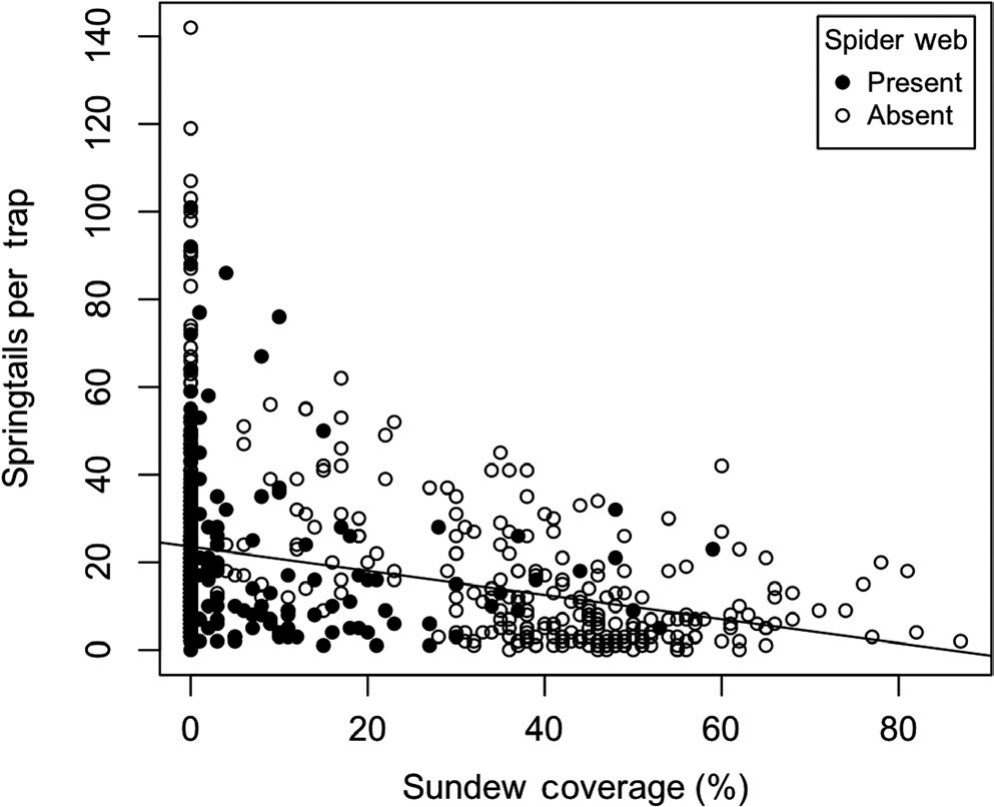
Number of springtails caught per sticky trap compared to sundew density as measured by the percentage of grids with sundews. Open circles indicate no sheet‐web spiders absent and solid circles indicate at least one sheet‐web present

**FIGURE 7:**
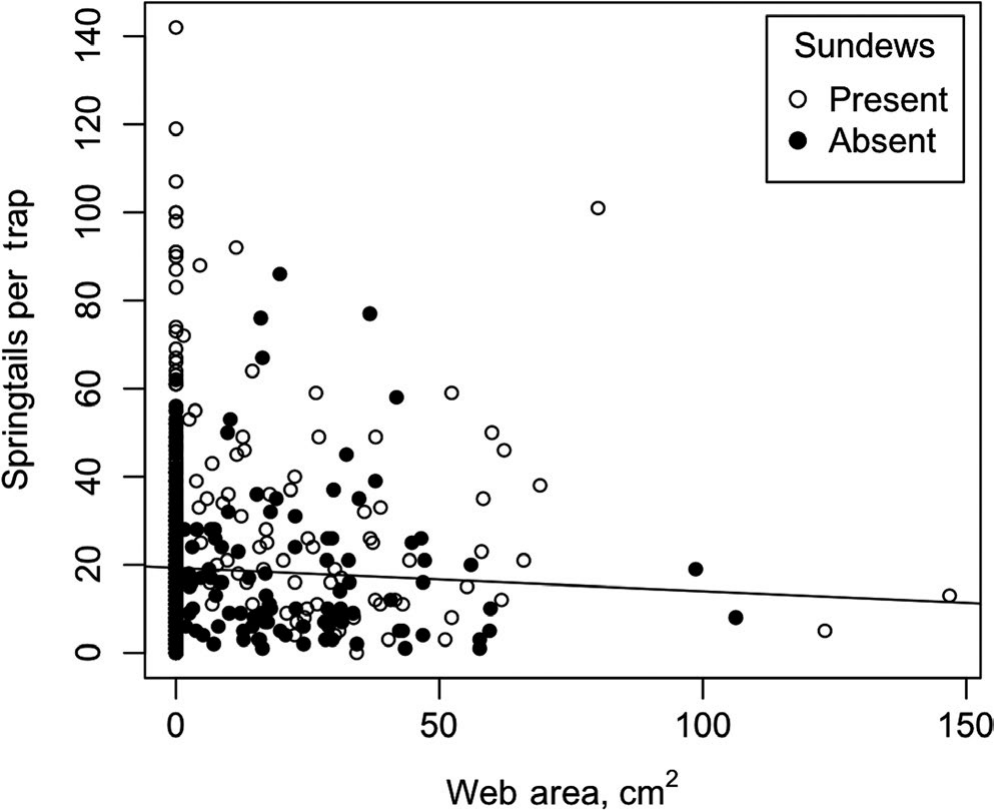
Number of springtails per sticky trap compared to the area of the associated sheet‐web. Open circles indicate sundews were present, while solid circles indicate sundews were absent

**FIGURE 8:**
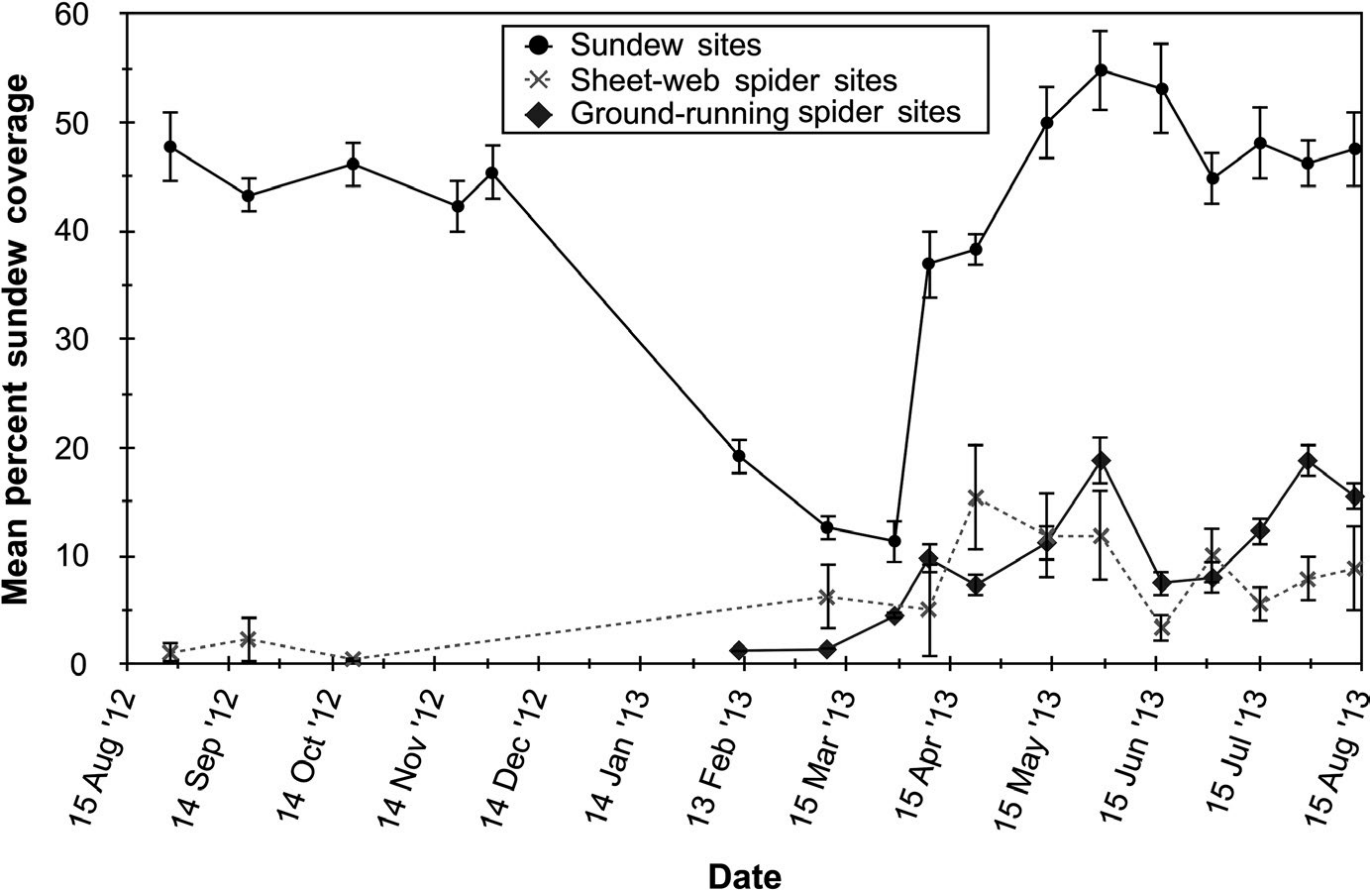
Number of springtails (±1 *SE*) caught on sticky traps placed sundew sites, spider sites, and control sites over 17 dates that sticky traps were places in the meadow

**FIGURE 9:**
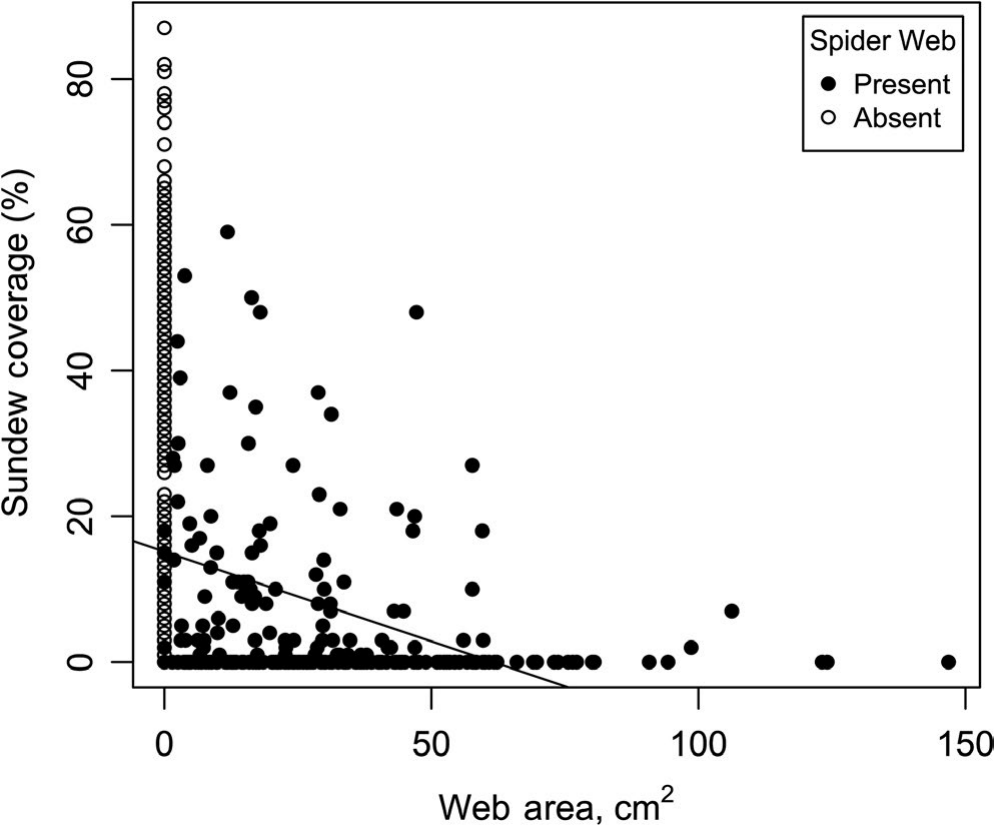
Percentage of sundew coverage compared to the area of sheet‐webs. Open circles represent sites where sundews where present and sheet‐webs were absent. These data points are also represented as 0 cm^2^ web area. Solid circles indicate sheet‐webs present

**FIGURE 10:**
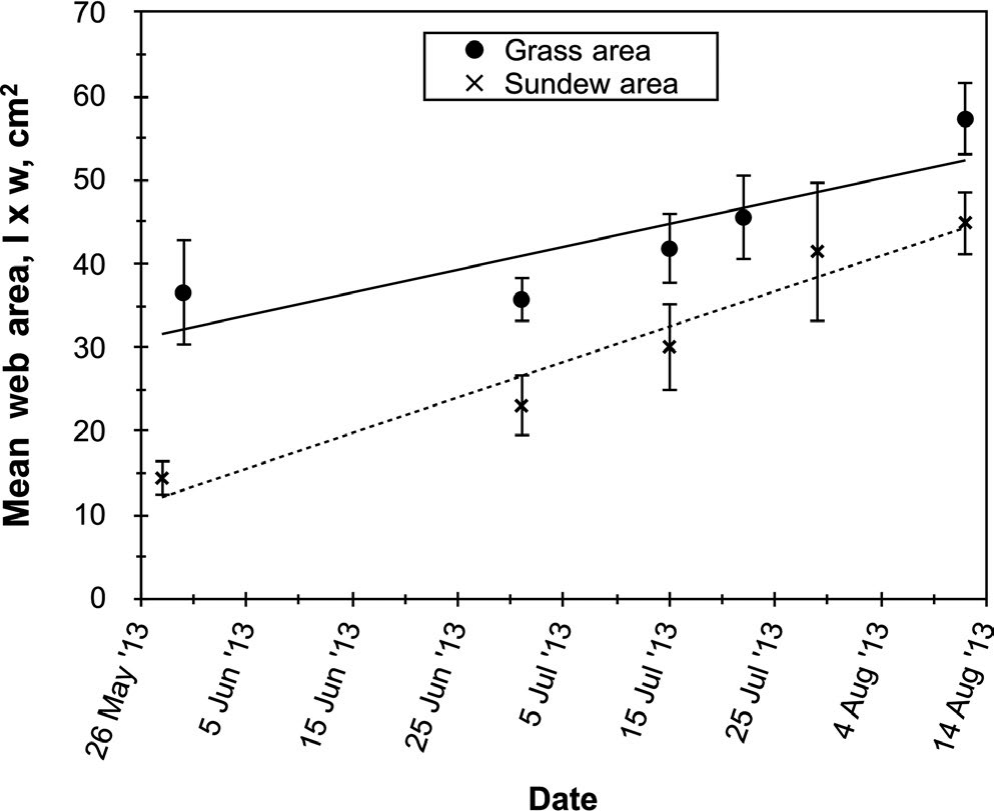
Mean sheet‐web area is compared to sites with grass lacking sundews to sites with sundews measured on nine dates from May to August 2013

The number of springtails caught on sticky traps placed at sundew, spider, and control sites differed significantly (*F*_2,734_ = 296.94, *p* < .001; Figure 4). Pairwise comparisons showed that sticky traps placed in sundew sites captured significantly fewer springtails than traps set in spider (*p* < .001) and control sites (*p* < .001), but there was no difference between spider and control sites (*p* > .05). This overall effect emerged despite a marked reversal for two winter samples when sundew numbers were greatly reduced. Comparison of sticky traps set out in open versus grass locations revealed greater numbers of springtails were captured in the open areas (*F*_1,50_ = 176.43, *p* < .001; Figure 5). Results of two general linear mixed‐effect models testing specific predictions (i–v) of competition are enumerated in Table 1 and summarized as follows: (i) The number of springtails captured per sticky trap declined significantly with greater sundew coverage (Figure 6), independent of the similarly negative effect of grass; (ii) springtail capture was not negatively affected by spider web presence; (iii) where sheet‐web spiders were present, though, springtail activity–density (=number of springtails per sticky trap) decreased as web area increased (Figure 7); (iv) spider web presence and ground‐running spider presence were highly significant predictors of sundew abundance. Sundews were significantly more abundant where spiders were absent (*F*_2,211_ = 493.47, *p* < .001; Figure 8). Sundew sites averaged 40.5% sundew cover, while spider sites averaged 6.9% sundew cover, and ground‐running spider sites averaged 8.7% sundew cover. Pairwise comparisons found no statistically significant difference in sundew cover between sites with the two spider types and highly significant differences between sundew sites and sheet‐web sites (*p* < .001) and ground‐running spider sites (*p* < .001); (v) there was a highly significant negative correlation between web area and percent sundew (Figure 9), helping to distinguish the effects of sundew abundance from the separate effect of grass. Sheet‐webs located in the open with sundews were significantly larger than webs located in the adjacent (within 5 m) grass areas with no sundews (*F*_1,45_ = 14.092, *p* < .001; Figure 10). Thus, sheet‐webs were smaller in the open area than in the grass, but within the open area, wherever sundew abundance was greater, they were smaller still.

**Table 1:** Fixed effect results for general linear mixed‐effects models testing our five predictions of competition (i, ii, iii, iv, v): Parameter estimates (*β*), standard error (*SE*), test statistic (*z*), and significance level (*p*) are reported for standardized independent variables

Independent variable	*β*	*SE*	*z*	*p*
Dependent variable = Springtails per sticky trap (*df* = 727)				
(Intercept)	2.68815	0.14461	18.589	<.001
(i) Sundews	−0.36582	0.01017	−35.988	<.001
(ii) Web	−0.01476	0.01248	−1.183	.2366
(iii) Web area	−0.03141	0.01422	−2.209	.0272
Grass	−0.15970	0.01807	−8.838	<.001
Dependent variable = Percent Sundew coverage (*df* = 981)				
(Intercept)	−0.15888	3.56419	−0.045	.964
(iv) Web	−0.31040	0.02034	−15.260	<.001
(v) Web area	−0.31086	0.03114	−9.982	<.001
(iv) Wanderer	−0.40285	0.03621	−11.126	<.001

Note

Sundews2 = percent sundew coverage, Web = sheet‐web presence, Web Area = sheet‐web length multiplied by width (cm^2^), Wanderer = Ground‐running spider presence, and Grass = open versus grass. Sampling date was a random effect to control for time.

### 4.5 Prey captured by sundews

A total of 224 arthropods were found attached to 150 sundew leaves. Of these, 91 (40.6%) were springtails, 88 (39.3%) were flies, and 29 (12.9%) were ants (3) and bees (26) (Order: Hymenoptera). The remaining 16 arthropods included five spiders, two mites, four aphids, two leafhoppers, two crickets (Order: Orthoptera), and one beetle (Order: Coleoptera). Sixty‐two (41.3%) of the individual sundew leaves examined had springtails trapped on their trichomes.

### 4.6 Direct spider–sundew interactions

Seventeen *N. agilis* were observed in the arena for a total of 62 min during which they made contact with sundews 221 times. On 16 occasions, spiders stopped when their front legs made contact with a sundew then turned away avoiding further contact. Spiders ran over sundews 205 times with mucilage attaching to their legs only 24 times. Of these, it took spiders 9.3 s on average to pull away from sundews. The 17 spiders averaged 2.9 mm in length and were smaller than the average sundew (4.7 mm dia) in the arena.

## 5 DISCUSSION

Sundew density as influenced by population size and plant diameter both fluctuated greatly over the study period and were influenced by precipitation, drought, fire, time of year, and seed germination. These seasonal fluctuations are typical at our study site and have been observed every season over the last 10 years (J.J.K. Krupa, personal observation). Springtails were the most common taxa that co‐existed with sundews and most frequently caught on sundew leaves. For most of the year, activity–density of springtails was low in patches where sundews were most dense in contrast to areas where sundews were sparse or lacking. Both sheet‐web and ground‐running spiders were found in areas where sundews were sparse and springtail activity–density high. Molecular analysis of the gut content of 360 spiders (both sheet‐web and ground‐running) revealed that 50.3% tested positive for springtail DNA, signifying recent consumption of this prey.

There was no evidence of intraguild predation of sundews on spiders during this study. Only 5 very small spiders were found attached to sundew leaves. These accounted for 2.2% of all prey captured by sundews. Furthermore, sheet‐web spiders (*N. agilis*) that were only 38% the size of the average sundew ran over sundews 93% of 221 encounters during the arena experiment indicating sheet‐web spiders were not being directly impacted by these plants. The much larger wolf spiders often were observed to run over sundews unimpeded during our field study.

The results of this study suggest that the plant–animal interaction between sundews and spiders is most likely exploitative competition. Sheet‐web and ground‐running spiders were common and ubiquitous in the meadow, yet least common where sundews were most dense. Furthermore, where sundews were dense, sticky traps showed lower activity–density of springtails. When sundews were dormant, springtail activity–density was high around these dense stands of sundews. Our results indicate sundews were drawing down springtail numbers. Thus, springtails may have been a limiting resource in the presence of sundews. Spiders responded by avoiding these areas.

Those sheet‐web spiders in the highest densities of sundews were more likely to lack springtails in their guts than those caught in other locations. Sheet‐web spiders produce semipermanent webs that are initially built small and gradually expand over time if the site is productive (Janetos, 1982). Spiders continually monitor the quality of their microhabitat and adjust silk output to match foraging success. This is referred to as the probe web hypothesis (Welch, Haynes, & Harwood, 2012). Thus, where sundews are dense and prey less abundant, smaller, newer sheet‐webs should occur. These spiders are more likely to move away once the foraging patch has been assessed to be of lower quality. Although not quantified during this study, we frequently found the smallest sheet‐webs near dense patches of sundews lacked spiders suggesting the webs were abandoned.

Spiders are generally considered to be food limited (Anderson, 1974; Wise, 1993). Our data suggest this because fewer springtails were captured by larger sheet‐webs where sundews were absent. By virtue of being near dense patches of sundews, fewer springtails occurred, which may have limited their availability to spiders. Spiders are mobile predators capable of assessing prey levels and selecting patches where prey is abundant (Harwood, Sunderland, & Symondson, 2001, 2003; Uetz et al., 1999). Spiders in our study were sit‐and‐wait predators (sheet‐web species) and active foragers (ground‐running species) both having the option to relocate although inherent risks are associated with website abandonment (Scharf, Lubin, & Ovadia, 2011). By being mobile, spider location and web size can be influenced by prey availability.

Darwin (1859) suggested that competition should be strongest between closely related species, thus implying competition between members of different kingdoms should be weak. Diamond (1987) stated that based on Darwin's suggestion, the more distantly related the taxa the more asymmetrical the competition should be. Diamond used as example situations when fishermen and sea birds compete for fish. Humans have not suffered costs from this competition, while sea birds have experienced mass starvation. Furthermore, Barnes (2003) argued the greater the taxonomic distance between competitors, the more likely one will displace the other. Asymmetrical competition should be most extreme between species of different kingdoms to the point that amensalism (species A has a competitive effect on species B, but species B has no effect on species A) occurs (Hochberg & Lawton, 1990). Amensalism may describe the interaction between sundews and spiders under natural conditions. The potential for this asymmetry exists because sundews only extract nutrients from prey for growth and reproduction and will not die without prey (Dore Swamy & Ran, 1971; Ellison & Gotelli, 2009; Millett, Jones, & Walron, 2003, while spiders will die without prey as they acquire both nutrients and energy from prey (Toft, 2013; Wilder, 2011).

Amensalism was not observed in two previous laboratory studies. In one study (Jennings et al., 2010), wolf spiders (*Rabidosa rabida*) reduced seed production of pink sundews (*Drosera capillaris*) when prey (small crickets) availability was low. In a second study (Jennings et al., 2016), spiders (*Sosippus floridanus*) and oak toads (*Anaxyrus quericicus*) confined in terraria competed with pink sundews causing changes in sundew growth and trichome density depending on density of prey (crickets). However, none of these animal predators could relocate. Thus, the question remains whether under natural conditions spiders have a negative impact on sundews or whether the interaction is as asymmetrical as our current field study suggests.

The forms of competition to most likely negatively impact sundews are with other plants. This is especially true for small species like dwarf sundews. Sundews, like most carnivorous plants, depend on disturbance, especially fire, to compete with other angiosperms. As larger, faster growing angiosperms outcompete sundews for space and sunlight, disturbance reduces the asymmetry of competition that is detrimental to sundews. Furthermore, intraspecific competition may also have a greater impact on sundews than from any plant–animal interaction. Those that grow in low densities may face less competition for prey such as flies and springtails, than those growing in dense patches.

## 6 CONCLUSIONS

Doing extended field observations on sundew–spider interactions when spiders can move unimpeded is essential to understanding the dynamics between these two wet meadow predators. Springtails were abundant prey at our study site, and they were consumed by sundews, sheet‐web spiders, and ground‐running spiders. Thus, the potential existed for competition between sundews and spiders albeit asymmetrical competition. Exploitative competition best describes the interaction between sundews and spiders since spiders avoid areas with high densities of sundews where they can draw down prey. However, it is uncertain whether under natural conditions, spiders can negatively impact sundews. Thus, the interkingdom competition observed during this study is not only asymmetrical but probably an example of amensalism with spiders having no effect on sundews. Whether spiders able to move freely can have a negative impact on sundews in nature will require further investigation and field experiments.

## CONFLICT OF INTEREST

None declared.

## AUTHOR CONTRIBUTION

**James J. Krupa:** Conceptualization (lead); Data curation (lead); Formal analysis (lead); Funding acquisition (lead); Investigation (lead); Methodology (lead); Project administration (lead); Resources (lead); Software (lead); Supervision (lead); Validation (lead); Visualization (lead); Writing‐original draft (lead); Writing‐review & editing (lead). **Kevin R. Hopper:** Conceptualization (supporting); Data curation (supporting); Formal analysis (lead); Investigation (supporting). **Samuel B. Gruber:** Data curation (supporting); Methodology (supporting). **Jason M. Schmidt:** Data curation (supporting); Investigation (supporting); Validation (supporting). **James D. Harwood:** Conceptualization (equal); Funding acquisition (supporting); Project administration (supporting); Resources (equal). 

## Data Availability

Data from this study are archived in the Dryad Digital Repository (https://doi.org/10.5061/dryad.crjdfn31p).
